# Autophosphorylation and Cross-Phosphorylation of Protein Kinases from the Crenarchaeon *Sulfolobus islandicus*

**DOI:** 10.3389/fmicb.2017.02173

**Published:** 2017-11-07

**Authors:** Qihong Huang, Qing Zhong, Joseph B. A. Mayaka, Jinfeng Ni, Yulong Shen

**Affiliations:** State Key Laboratory of Microbial Technology, Shandong University, Jinan, China

**Keywords:** archaea, *Sulfolobus islandicus*, protein phosphorylation, protein kinase, cross-phosphorylation, regulatory network

## Abstract

Protein phosphorylation, one of the most important post-translational modifications, regulates almost every cellular process. Although signal transduction by protein phosphorylation is extensively studied in Eukaryotes and Bacteria, the knowledge of this process in archaea is greatly lagging behind, especially for Ser/Thr/Tyr phosphorylation by eukaryotic-like protein kinases (ePKs). So far, only a few studies on archaeal ePKs have been reported, most of which focused on the phosphorylation activities *in vitro*, but their physiological functions and interacting network are still largely unknown. In this study, we systematically investigated the autophosphorylation and cross-phosphorylation activities of ePKs from *Sulfolobus islandicus* REY15A using proteins expressed in *Escherichia coli* or *S. islandicus. In vitro* kinase assay showed that 7 out of the 11 putative ePKs have autophosphorylation activity. A protein Ser/Thr phosphatase, SiRe_1009, was able to dephosphorylate various autophosphorylated ePKs, confirming that these proteins are Ser/Thr kinases. Two ePKs, SiRe_2030 and SiRe_2056, homologs of typical eukaryotic PKs involved in peptide synthesis in response to various cellular stresses, exhibit highly efficient phosphorylation activities on both themselves and other ePKs. Overexpression of the protein kinases *in vivo* revealed that elevated level of either SiRe_1531 or SiRe_2056 inhibited the cell growth of *S. islandicus* cells. Finally, a phosphorylation network of the protein kinases was proposed and their putative physiological roles were discussed.

## Introduction

Protein phosphorylation is a reversible post-translational modification that regulates almost all cellular processes, such as cell cycle progression, cell mobility, DNA replication and repair (Humphrey et al., 2015). Abnormal protein phosphorylation/dephosphorylation in human cells is frequently either a driver or direct consequence of many diseases. Therefore, many protein kinases and phosphor-sites were designed as the targets of medicines (Cohen, 2002). Although protein phosphorylation has been extensively investigated in eukarya, the regulation mechanisms are still far from clear due to the presence of large amount of protein kinases/phosphatases (at least 518 protein kinases and 156 protein phosphatases in human) and their complex regulatory networks (Manning et al., 2002; Shi, 2009).

Protein kinases in bacteria were discovered later than those in eukaryotes. A number of previous studies in bacteria focused on histidine kinases in two-component systems, which was considered as the main regulatory phosphorylation in bacteria (Casino et al., 2010). The system contains two components, a sensor histidine kinase and a response regulator. The activated forms of the former would specifically recognize and phosphorylate the latter (Podgornaia and Laub, 2013). This linear signal transfer is different from the network regulatory pattern in eukaryotes. However, genome sequencing and proteomic studies have revealed that there are many eSTK in bacteria (Pereira et al., 2011). Recent studies on the eSTKs from *Mycobacterium tuberculosis* and the eSTKs and bacterial tyrosine kinases from *Bacillus subtilis* revealed that bacterial protein kinases exhibited both autophosphorylation and cross-phosphorylation activities on Ser, Thr, and Tyr residues. It seems that bacteria also have a regulatory network and/or cascades of protein kinases as those in eukaryotes. (Baer et al., 2014; Shi et al., 2014).

All sequenced archaeal genomes encode eukaryotic protein Ser/Thr/Tyr kinases and phosphatases, although the numbers are much fewer than those in eukaryotes (Kennelly, 2003), while histidine kinases are mainly found in euryarchaeota (Spudich and Stoeckenius, 1980). Archaeal ePKs contain most conserved subdomains of typical PKs. A number of studies on archaeal ePKs were performed in Crenarchaeota, especially in *Sulfolobus* (Lower and Kennelly, 2003; Albers and Driessen, 2005; LaRonde-LeBlanc et al., 2005; Hecker et al., 2009; Haile and Kennelly, 2011; Ray et al., 2015; Haurat et al., 2017; Hoffmann et al., 2017). In *Sulfolobus solfataricus*, there are 1318 phosphorylated sites in 540 proteins in total (Esser et al., 2012). These proteins contain most (21/26) clusters of orthologous groups, indicating that archaeal protein phosphorylation participates in most biological processes (Makarova et al., 2007). *Sulfolobus* cells possess at least two protein phosphatases. Deletion of saci_pp2a, one of the two phosphatase resulted in pronounced alterations in growth, cell shape and cell size (Reimann et al., 2013). The expression of the genes encoding components of motility system, the respiratory chain and transcriptional regulators, and their phosphorylation levels significantly changed in the deletion mutants (Reimann et al., 2013). In addition, it was revealed that the SIRV2 virus could not infect the *S. solfataricus* strain with a mutation in an ePK gene, *SsoPK2* (Deng et al., 2014), whereas deletion of the SsoPK2 homolog gene in *S. acidocaldarius* resulted in deficiency of pili formation (Henche et al., 2012). A report on a typical ePK from *S. acidocaldarius*, Saci1193, showed that the protein stimulated the expression of archaella genes probably by phosphorylating two negative regulatory proteins: ArnA and ArnB (Reimann et al., 2012). Very recently, deletion of either *Saci1193* or *Saci1181*, another ePK gene, resulted in reduced cell motility, while deletion of ePK gene *Saci1694* led to hypermotility. *Saci1193* and *Saci1181* are upregulated during starvation whereas *Saci1694* is constitutively expressed. Both Saci1193 and Saci1694 phosphorylated ArnB at its C-terminus (Haurat et al., 2017; Hoffmann et al., 2017). These studies suggested that the ePKs play complex regulatory roles in controlling the expression of archaellum components. The limited functional studies of ePKs mainly focus on the regulation of pili and archaella formation. However, other potential roles of the ePKs and the physiological functions of other archaeal ePKs are largely unknown.

The archaeal ePKs seem to harbor a hierarchy of regulatory networks since they exhibit the characteristics of eSTKs. All typical protein kinases (Hanks-type kinases) contain a conserved catalytic domain folding into two lobes, a smaller amino-terminal lobe (N-lobe) and a larger carboxy-terminal lobe (C-lobe) connected by a hinge region (Hanks and Hunter, 1995). The domain is further divided into 12 subdomains. There are several important residues for catalytic activity: the Lys residue in subdomain II; the conserved Asp in the subdomain VIb, which is likely to be the catalytic base involved in the phosphotransfer reaction, as well as the invariant Asp in subdomain VII, which functions in the orientation and anchoring of the ATP (Hanks and Hunter, 1995; Hanks, 2003). It appears as a common mechanism in eukaryotes that Ser or Thr residues in the activation loop of PKs, a conserved peptide bordered by the subdomains VII and VIII, is phosphorylated to activate PKs’ activities (Nolen et al., 2004). It was shown that several *Sulfolobus* ePKs contained the activation loop, indicative of a kinase cross-talk similar to that in eukaryotes and bacteria (Esser et al., 2016). So, it is interesting to unveil the hierarchy of archaeal ePKs regulatory networks.

In this study, we systematically purified and analyzed eleven putative ePKs from *S. islandicus*. Their autophosphorylation and cross-phosphorylation activities were investigated. The effects of ePK overexpression on *Sulfolobus* cell growth were also analyzed. Based on both *in vitro* and *in vivo* results, we propose a framework of the phosphorylation network of the protein kinases. Their physiological roles of these protein kinases were also discussed.

## Materials and Methods

### Strains and Growth Conditions

*Sulfolobus islandicus* strain E233S (Δ*pyrEF* Δ*lacS*, **Table 1**) (hereafter E233S) was grown at 75°C in the mineral salt medium supplemented with 0.2% (wt/vol) sucrose (S), 0.2% (wt/vol) tryptone (T), a mixed vitamin solution (V), and 0.01% (wt/vol) uracil (U) (named MTSVU medium), as described previously (Deng et al., 2009). MSV medium supplemented with 0.2% casamino acid (C) was used for cultivating uracil prototrophic strains. MTV medium supplemented with 0.2% arabinose (A) was used for protein expression. Phytagel (0.8% [wt/vol]) was added in the medium for making plates.

**Table 1:** *Sulfolobus* strains used in this study.

Strains	Genotype	Source
*Sulfolobus islandicus* REY15A (E233S)	Δ*pyrEF*Δ*lacS*	Deng et al., 2009.
E233S/pSeSD-0101KD-C-His, E233S/pSeSD-0171-C-His, E233S/pSeSD-0181-C-His, E233S/pSeSD-1057-C-His, E233S/pSeSD-1531-C-His, E233S/pSeSD-1570-C-His, E233S/pSeSD-1639-C-His, E233S/pSeSD-1810-C-His, E233S/pSeSD-2030-C-His, E233S/pSeSD-2056KD-C-His, E233S/pSeSD-2600-C-His	E233S with various protein kinase genes on the expression vector pSeSD harboring *araS* promoter coding for C-His-tagged protein kinases	This work

### Plasmids Construction

To construct the plasmids for expressing N-His-tagged protein kinases or protein phosphatases in *Escherichia coli*, each gene (or a gene fragment containing kinase domain) was amplified by PCR using their corresponding primers (*Nde*I-F/*Sal*I-R) listed in Supplementary Table S1. The *Nde*I restriction sites in *SiRe_0181, SiRe_2056, SiRe_2600*, and *SiRe_0241* genes were mutated using splicing by overlap extension (SOE) PCR. The PCR product of each gene was digested and ligated into the *Nde*I and *Sal*I sites of the pET15b vector. For expression of inactive protein kinases in *E. coli*, the conserved Asp or Glu within the core catalytic motif of each gene was mutated by SOE PCR using the wild type gene as template and inserted into pET15b.

The vectors for overexpression of C-His-tagged protein kinases in *S. islandicus* were constructed by amplification of each gene (or kinase domain) using their corresponding primers (*Nde*I-F/Nostop-*Sal*I-R) and insertion of the gene fragment into the *Nde*I and *Sal*I sites of the vector pSeSD (Supplementary Table S2) carrying the *pyrEF* marker (Peng et al., 2012), yielding kinase overexpression vector. The primers used for PCR are listed in Supplementary Table S1.

### Transformation of *S. islandicus* Strains and Determination of the Growth

The expression plasmids were transformed into *S. islandicus* cells by electroperation as previously described (Deng et al., 2009). To obtain growth curves, cells were grown to early log-phase and transferred for 3–4 times before the measurement. The initial OD_600_ value was 0.03–0.04 and the ODs were measured every 6 or 12 h. A growth curve was made based on the data from at least three parallel experiments.

### Western Blot Analysis

Two milliliters of the cultures in early log-phase was collected by spinning down. The cells were resuspended in 40 μl of a buffer containing 50 mM Tris-HCl pH 8.0, 100 mM NaCl and 10 μl of 5 × SDS-PAGE loading buffer. The mixture was boiled for 10 min and loaded onto a SDS-PAGE gel. The proteins in the PAGE gel were transferred onto a PDVF membrane. The membrane was incubated with anti-6 × His antibodies and HRP-labeled goat anti-mouse IgG under standard Western blot conditions. The image was obtained by ImageQuant 400 (GE Healthcare, Buckinghamshire, United Kingdom).

### Protein Purification

To purify wild type and mutant protein kinases from *E. coli*, the protein expression was induced at either 37°C for 4 h or 16°C for 12–16 h. The cell pellets were resuspended in buffer A (50 mM Tris pH 8.0 or 9.0, 200 mM NaCl, and 5% glycerol) and lysed by sonication. The soluble proteins were heated at 70°C for 30 min and the supernatants after centrifugation were loaded onto a Ni-NTA column pre-equibilirated with buffer A. Unbound proteins were washed by wash buffer (buffer A supplemented with 40 mM imidazole) and target proteins were eluted by elute buffer (buffer A supplemented with 250 mM imidazole). The eluted fractions containing target proteins were pooled and concentrated. The proteins were subsequently purified by gel filtration using a Superdex^TM^ 200 10/300 column (GE Health, United Kingdom) in the corresponding buffer. Fractions containing the purified proteins were collected, aliquoted, and stored at -80°C after frozen with liquid nitrogen. The protein concentration was determined by the Bradford method with bovine serum albumin as the standard. The procedure for purification of SiRe_0101KD-C-His from *S. islandicus* was the same as above except that the protein was induced by arabinose at 75°C for 12 h and purified without heat-treatment. The fractions containing SiRe_0101KD-C-His were analyzed by SDS-PAGE and the whole lane was cut for mass spectrometry analysis by BGI (Beijing Protein Research Center Co., Ltd.).

### *In Vitro* Kinase Assay

To analyze the autophosphorylation activities of protein kinases, a certain mount (1 or 2 μM as specified) of wild type protein kinase was added into a reaction mixture (20 μl) containing 25 mM Tris-HCl pH 8.0, 50 mM NaCl, 5 mM MgCl_2_ (or MnCl_2_), 2 mM DTT, 4.2 nM [γ-^32^P]ATP (111 TBq/mmol, PerkinElmer), and 50 μM cold carrier ATP. The mixture was incubated at 65°C for 30 min and the reaction was stopped by adding 5 × SDS-PAGE loading buffer and boiling for 10 min. The samples were analyzed by 12% or 15% SDS-PAGE. The autoradiographs were quantified by the software ImageQuant 5.2.

For detecting the cross-phosphorylation activities between two protein kinases, the reaction is the same as above except that an inactive protein kinase (2 μM) was added as the substrate. The dephosphorylation activities of the protein phosphatases were analyzed by adding a protein phosphatase (2 μM) in the autophosphorylation reaction above.

## Results

### Bioinformatics Analysis and Purification of *S. islandicus* Eukaryotic-Like Protein Kinases

**FIGURE 1:**
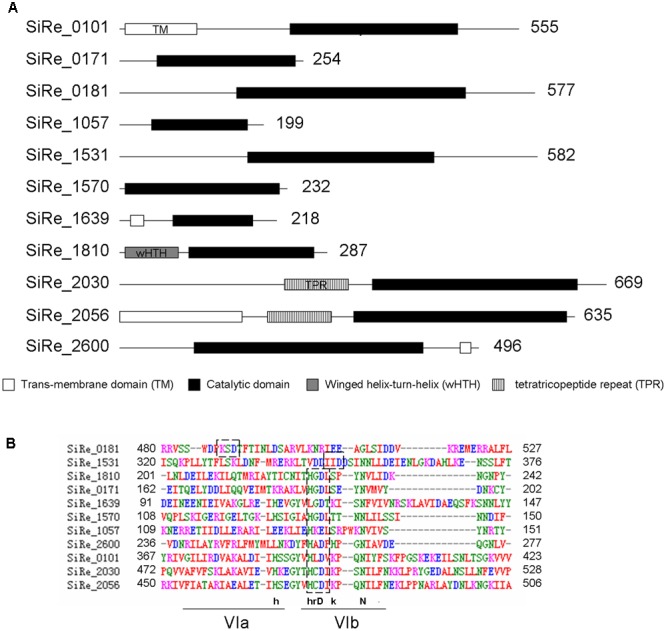
Putative eukaryotic-like protein kinases (ePKs) in *Sulfolobus islandicus* REY15A. **(A)** Domain organization of the protein kinases. The domains were predicted according to database of NCBI (https://www.ncbi.nlm.nih.gov/) and UniProt (http://www.uniprot.org/) and are shown in boxes with the amino acid numbers being indicated on the right. **(B)** Alignment of the core catalytic domains of the predicted ePKs. The sequences were analyzed by ClustalW. The conserved HRD motif within subdomain VIb is indicated in dashed boxes. The HRD motif in SiRe_0181 and SiRe_1531 are identified according to the studies on their homologs (Sso2387 and Sso0469, respectively) from *S. solfataricus* (Lower and Kennelly, 2003; Lower et al., 2004). The consensus sequences of the motifs are depicted above the lines: uppercase, universally conserved amino acid residues; lowercase, highly conserved amino acid residues.

There are ten potential ePKs encoded in the *S. solfataricus* genome (Kennelly, 2014). As *S. islandicus* has highly close phylogenetic relationship with *S. solfataricus*, the homologs of these 10 ePKs (SiRe_0101, SiRe_0171, SiRe_0181, SiRe_1057, SiRe_1531, SiRe_1570, SiRe_1810, SiRe_2030, SiRe_2056, and SiRe_2600) were all found in *S. islandicus* REY15A by BLAST analysis (**Table 2**). In addition, SiRe_1639 is also annotated as a Mn^2+^-dependent serine/threonine protein kinase containing a protein kinase catalytic domain (KD) according to the genomic information of *S. islandicus* REY15A (**Figure 1A**) (Guo et al., 2011). All these 11 ePKs contain a putative conserved Asp residue (or Glu for SiRe_1057, see below) at the HRD motif within the catalytic domain (subdomain VIb) as revealed by sequence alignment (**Figure 1B**) (Hanks and Hunter, 1995). The Asp sites in SiRe_0181 and SiRe_1531 were identified previously by sequence analysis (Sso2387 and Sso0469 in *S. solfataricus*, respectively). Several ePKs also harbor other domains that may facilitate their functions in the cell, such as TM domain, wHTH, and TPR (**Figure 1A**).

**Table 2:** *Sulfolobus islandicus* protein kinase homologs in other *Sulfolobus* species.

*S. islandicus*	*S. solfataricus*	*S. acidocaldarius*	*S. tokodaii*
SiRe_0101	Sso2291	–	–
SiRe_0171	Sso2374	Saci_0965	STK_05130
SiRe_0181	Sso2387	Saci_2317	STK_05220
SiRe_1057	Sso1038	Saci_1289	STK_09530
SiRe_1531	Sso0469	Saci_0435	STK_01810
SiRe_1570	Sso0433	Saci_0850	STK_03640
SiRe_1639	Sso0361	Saci_1477	STK_13820
SiRe_1810	Sso0197	Saci_0796	STK_02330
SiRe_2030	Sso3207	Saci_1193	STK_08100
SiRe_2056	Sso3182	–	–
SiRe_2600	Sso2605	Saci_1664	STK_16520

**FIGURE 2:**
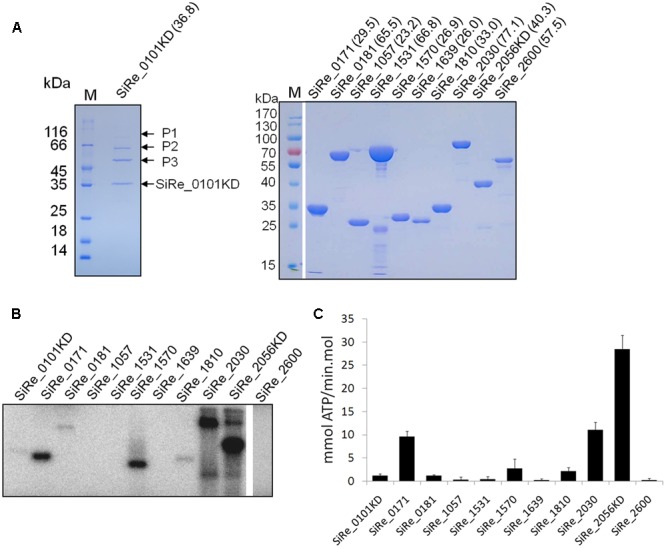
Purification of the eukaryotic-like protein kinases (ePKs) of *S. islandicus* REY15A and analysis of their autophosphorylation activities. **(A)** Purification of His-tagged ePKs expressed in *Escherichia coli* (right) or *S. islandicus* REY15A (left). The predicted molecular weight (kDa) are shown in the parentheses. M, molecular size marker. P1, P2, and P3, proteins co-purified with SiRe_0101KD. **(B)** Autophosphorylation activities of *S. islandicus* ePKs. Each ePK (2 μM) was incubated in the reaction mixture containing 4.2 nM [γ -^32^P]ATP and 50 μM cold carrier ATP (see Materials and Methods) at 65°C. The samples were analyzed by 15% SDS-PAGE. The experiments were performed for at least three times for each enzyme. Representative images of ePKs autophosphorylation activities are shown. **(C)** The autoradiographs in B are quantified by ImageQuant. The data were obtained from three independent experiments. The bars indicate standard deviation.

To investigate the phosphorylation network of these ePKs, the proteins were expressed and purified from *E. coli*. Initially, all ePK proteins were expressed but the expressed proteins were mostly, or at least partially, insoluble during cell lysis by sonication (data not shown). After optimizing the conditions for protein induction and purification, we were able to get the full length of the majority of these proteins except for SiRe_0101 and SiRe_2056 (**Figure 2A**). Since both ePKs contain a TM domain at their N-terminal which would probably affect their expression in *E. coli*, we then attempted to purify the catalytic domains (KDs) of both ePKs. However, only soluble SiRe_2056KD was obtained using *E. coli* expression system. The expression of SiRe_0101 was further tried and the protein was successfully expressed in *S. islandicus* using shutter vector pSeSD (Peng et al., 2012). Western blot analysis showed that the band of SiRe_0101KD, but not the full length, was visible after purification by Ni-NTA column (Supplementary Figure S1). There were also several other proteins in the fractions (9–11 ml) containing SiRe_0101KD even after purification by gel filtration (**Figure 2A**).

### Autophosphorylation Activities of Various *S. islandicus* ePKs and Their Dephosphorylation by Two Protein Phosphatases

Autophosphorylation is the dominant mode of ePK activation and a mechanism of efficient signal amplification (Nolen et al., 2004). It was shown that several *S. solfataricus* and *S. acidocaldarius* ePKs exhibited autophosphorylation activities mainly on Ser or Thr (Lower and Kennelly, 2003; Albers and Driessen, 2005; LaRonde-LeBlanc et al., 2005; Hecker et al., 2009; Haile and Kennelly, 2011; Ray et al., 2015; Haurat et al., 2017; Hoffmann et al., 2017). To systematically analyze the autophosphorylation activities of *S. islandicus* ePKs, each ePK was incubated with [γ-^32^P]ATP in a reaction mixture for the kinase assay. The results revealed that, among these ePKs, seven ePKs (SiRe_0101KD, SiRe_0171, SiRe_0181, SiRe_1570, SiRe_1810, SiRe_2030, and SiRe_2056KD) exhibited autophosphorylation activities under our experimental conditions with SiRe_2056KD having the highest activity (**Figures 2B,C**). In addition, SiRe_2030 exhibited higher autophosphorylation activity in the presence of Mn^2+^ than Mg^2+^, while the activity of SiRe_1570 was very low in the presence of Mg^2+^ (Supplementary Figure S2). Hereafter, MnCl_2_ was added into the kinase assay mixture for SiRe_1570 and SiRe_2030 in subsequent analysis. The quantitative result showed that SiRe_2056KD was able to incorporate 28 mmol phosphate/min⋅mol into itself, which is almost the same as its homolog in *S. solfataricus* (Ray et al., 2015).

**FIGURE 3:**
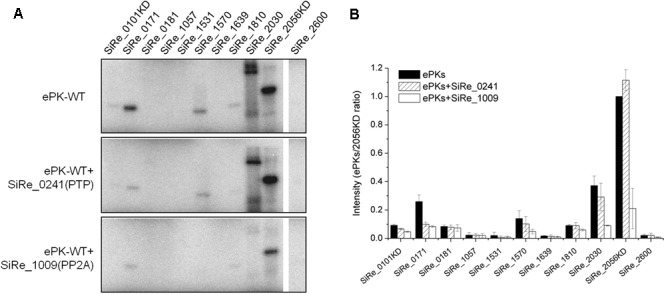
Dephosphorylation of the autophosphorylated *S. islandicus* ePKs by two protein phosphatases (PPs), SiRe_0241 (protein tyrosine phosphatase, PTP) and SiRe_1009 (phospho-Ser/Thr phosphatase, PP2A). **(A)** Representative profiles of the phosphorylation (upper panel) and dephosphorylation reactions (middle and lower panels). For the dephosphorylation reactions, each ePK (1 μM) was incubated with one of the two PPs in a reaction at 65°C and the samples were analyzed by 12% SDS-PAGE. The experiments were performed for at least three times. Representative images of ePKs autophosphorylation (upper panel), dephosphorylation by SiRe_0241 (middle panel) and SiRe_1009 (lower panel) are shown. **(B)** Quantitative results of the autoradiographs in **(A)**. The signals of each ePK are normalized to the autophosphorylation signal of SiRe_2056KD. The bars indicate standard deviation.

To confirm that the autophosphorylated residues were Ser/Thr or Tyr, two protein phosphatases (SiRe_0241 and SiRe_1009) were purified from *E. coli* and applied in the dephosphorylation assay (Supplementary Figure S3). SiRe_0241 belongs to the family of PTP, while SiRe_1009 is a phosphor-Ser/Thr phosphatase (PP2A, a subfamily of PPP). It was shown that Saci-PTP displayed a phosphatase activity toward both pTyr and pSer/pThr with a much higher activity on pTyr (30- and 131-fold higher as compared with those on pSer and pThr, respectively), but Sso-PTP exhibited phosphohydrolase activity only toward pTyr (Chu and Wang, 2007; Reimann et al., 2013). A phylogenetic analysis on PPPs from three domains of life showed that archaeal PPPs were inherited from the last universal common ancestor (Kennelly, 2014). So far, the studies on archaeal PPPs, including Saci-PP2A, revealed that they all exhibited specific pSer/pThr activity (Solow et al., 1997; Mai et al., 1998; Reimann et al., 2013). Because high amino sequence conservation between SiRe_0241 and Sso-PTP as well as SiRe_1009 and Saci-PP2A, we assume that SiRe_0241 and SiRe_1009 are PTP and PP2A in *S. islandicus*, respectively. In our dephosphorylation assay (**Figure 3**), SiRe_0241 (Sis-PTP) only slightly reduced the signal strength, whereas the bands corresponding to all the autophosphorylated proteins were nearly invisible or significantly decreased in the presence of SiRe_1009 (Sis-PP2A). Collectively, our results suggested that most ePKs phosphorylated themselves on Ser/Thr sites. The results are consistent with those of the phosphoproteomics analysis of the SiRe_0171 and SiRe_2030 homologs in *S. acidocaldarius* showing that both ePKs were phosphorylated mainly on Ser/Thr sites *in vivo* (Reimann et al., 2013).

### Cross-Phosphorylation Relationship among ePKs

**FIGURE 4:**
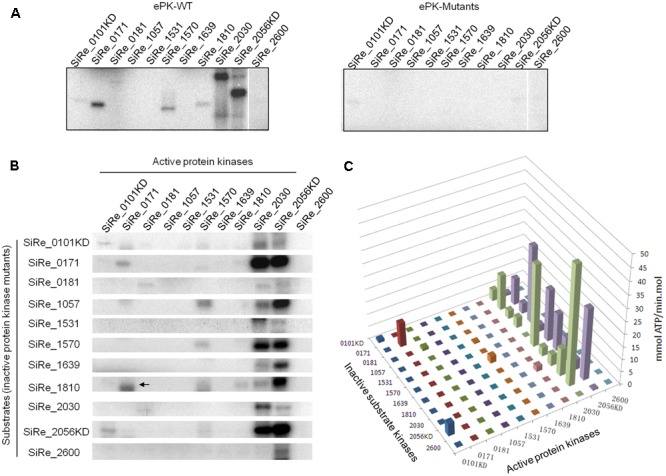
Cross-phosphorylation activities among various ePKs. **(A)** Autophosphorylation activities of the wild type (WT, left panel) and mutants (M, right panel) ePKs. **(B)** Cross-phosphorylation activities of each ePK on eleven inactive ePK mutants. Each ePK (2 μM) was incubated with the same concentration of each of the eleven inactive ePKs in the reaction containing 4.2 nM [γ -^32^P]ATP and 50 μM cold carrier ATP. The signals on the diagonal indicate autophosphorylation. Off-diagonal bands in each column reflect cross-phosphorylation. The arrow indicates the autophosphorylation signal of wild type SiRe_0171 whose size (30 kDa) is a bit smaller than that of SiRe_1810 (33 kDa). The experiments were performed at least for three times. Representative gel images are shown. **(C)** Quantitative analysis of the auto/cross-phosphorylation activities in **(B)**. The data were obtained from three independent experiments.

The complexity of eukaryotic regulation by protein phosphorylation is due to their complicated cross-phosphorylation network of protein kinases and signaling cascade (Breitkreutz et al., 2010). Recent studies in *M. tuberculosis* and *Bacillus subtilis* indicated that the behavior of bacterial ePKs network was also similar to that in eukaryotes (Baer et al., 2014; Shi et al., 2014). To understand the cross-talk between various ePKs from *S. islandicus*, inactive ePKs were generated by introducing a mutation (Asn or Ala) at the conserved Asp or Glu site (for SiRe_1057) within the catalytic domain of wild type ePKs (**Figure 1B** and Supplementary Figure S4). Glu residue in SiRe_1057 is highly conserved in all analyzed archaeal homologs (Supplementary Figure S4). As shown in **Figure 4A**, all site-directed mutants except for SiRe_0181D490N did not have autophosphorylation activity. The Asp within the HRD motif of SiRe_0181 was identified according to the studies on the *S. solfataricus* homologs (Sso2387) which is annotated as a secretion ATPase (Lower and Kennelly, 2003; Albers and Driessen, 2005). However, after substitution of Asp by Asn, the mutant protein still exhibited autophosphorylation activity (Supplementary Figure S5). It seems that the mechanism of SiRe_0181 autophosphorylation was different from other PKs and the conserved Walker A and Walker B motifs may be essential for the activity. To test this, we constructed another SiRe_0181 mutant by replacing Lys in Walker A motif with Ala (SiRe_0181K324A) and examined its kinase activity. The result showed that SiRe_0181K324A did not phosphorylate itself (Supplementary Figure S5). This mutant was used for the subsequent cross-phosphorylation assay.

The cross-phosphorylation activities among various pairs of ePKs were then analyzed using complete set of active and inactive ePKs. We found that SiRe_2030 and SiRe_2056KD were the only two ePKs having highly efficient phosphorylation activity on heterologous ePKs. SiRe_2056KD was able to phosphorylate all other ten ePKs, whereas SiRe_2030 phosphorylated most ePKs except for SiRe_1531 and SiRe_2600 (**Figures 4B,C**). Interestingly, SiRe_0101KD only phosphorylated SiRe_2056KD, while SiRe_1570 had low activity on SiRe_1057. Other ePKs did not phosphorylate heterologous ePKs regardless of whether they had autophosphorylation activities or not. Taken together, SiRe_2030 and SiRe_2056 may serve as the active ePKs that respond to different cellular stresses *in vivo*, while the others (excluding SiRe_0101) might be the substrate ePKs.

### Effect of ePKs Overexpression on the Growth of *S. islandicus*

**FIGURE 5:**
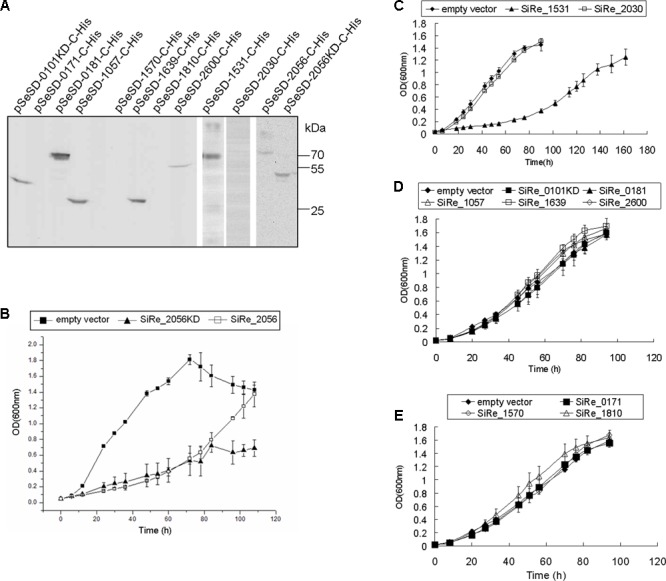
The effects of ePKs overexpression on the growth of *S. islandicus* REY15A. **(A)** Western blot analysis of ePK (or ePK-KD) expressed in *S. islandicus*. The samples were taken from cultures at mid-log phase (OD ∼ 0.4–0.6) for Western blot using anti-His-tag antibody. Protein size markers are indicated on the right. **(B–E)** Growth curves of each ePK (or ePK-KD) overexpression strain. The OD values were measured every 6 or 12 h. The growth curves were obtained from three independent cultures. The bars indicate standard deviation.

The analysis of gene overexpression phenotypes provides a unique way to study gene functions, because it can lead to hyper-effects on cells often due to mis-regulation and those strictly regulated in the cell could not be overexpressed (Stevenson et al., 2001; Boyer et al., 2004). To analyze the importance of ePKs in *S. islandicus*, overexpression strains of each ePK were constructed with pSeSD-based vectors and the C-terminal his-tagged proteins were induced by adding arabinose into the medium. It is confirmed that the pSeSD-based plasmids carrying each kinase gene was maintained in the *Sulfolobus* cell by plasmid re-extraction and digestion (data not shown). Seven ePKs or their KDs (SiRe_0101KD, SiRe_0181, SiRe_1057, SiRe_1639, SiRe_2600, SiRe_1531, and SiRe_2056) were overexpressed as detected by Western blot analysis against the His-tag, while the expression of SiRe_0171, SiRe_1570, SiRe_1810, and SiRe_2030 were not detectable (**Figure 5A**). However, only strains overexpressing SiRe_1531 and SiRe_2056 overexpression showed growth retardation (**Figures 5B,C**). The other strains showed no difference in growth from that of the control carrying empty vector (**Figures 5C–E**). The strains with pSeSD carrying SiRe_0171, SiRe_1570, SiRe_1810, or SiRe_2030 genes grew normally probably because these ePKs were not overexpressed. The protein levels may be repressed by either transcriptional regulation or protein degradation. The reasons why these kinases could not be overexpressed need further investigation. In addition, expression of SiRe_2056KD also resulted in cell growth retardance.

### Mass Spectrometry Analysis of Proteins Co-purified with SiRe_0101KD from *S. islandicus*

As we mentioned above, in the fractions of SiRe_0101KD purified from *S. islandicus* by gel filtration following Ni-NTA column purification, several other protein bands were clearly visible on the SDS-PAGE gel. To identify these proteins and provide hints of potential functions of SiRe_0101, the whole lane was cut and the protein identity was analyzed by mass spectrometry. More than 100 proteins were identified (Supplementary Table S3). Among those with high score, there were a number of proteins putatively involved in protein folding and degradation, including thermosome (SiRe_1214[α], SiRe_1716[β], and SiRe_2245[γ]), AAA family ATPase of CDC48 subfamily (SiRe_1582 and SiRe_1832), the subunits of proteasome endopeptidase complex (SiRe_1271[α], SiRe_1720[β1], and SiRe_1237[β2]), heat shock protein Hsp20 (SiRe_0216), and prefoldin subunits (SiRe_1279[α] and SiRe_1653[β]). According to the protein masses and scores, the three visible bands P1, P2, and P3 might be Cdc48 (SiRe_1582, 85.5 kDa), thermosome (SiRe_1214 or SiRe_1716, 59.7 kDa), and TIP49 (SiRe_0238, 50.1 kDa), respectively (**Figure 2A**). Two ePKs, SiRe_0181 and SiRe_1639, were also found in the fraction, as well as other proteins involved in DNA metabolism, translation, and energy production, amino acid transport and metabolism. The relationship of SiRe_0101 with Cdc48, the thermosome, and TIP49 needs further investigation.

## Discussion

Studies on archaeal protein kinases are limited and the majority mainly focused on their *in vitro* biochemical activities. For the first time, we systematically purified and analyzed the autophosphorylation and cross-phosphorylation activities of eleven putative *S. islandicus* ePKs. We found that SiRe_0101KD, SiRe_0171, SiRe_0181, SiRe_1570, SiRe_1810, SiRe_2030, and SiRe_2056KD had autophosphorylation activities and SiRe_2030 and SiRe_2056KD exhibited higher activities either on themselves or on most heterologous ePKs than the others. Most eukaryotic PKs which are referred to as RD kinases contain a conserved Arg located adjacent to the key catalytic residue Asp (Krupa et al., 2004). This positively charged Arg inhibits catalysis by the neighboring negatively charged Asp. The inhibition can be removed by phosphorylation of the activation loop which produces negatively charged phospho-amino acids and neutralize the positively charged Arg, resulting in kinase activation (Johnson et al., 1996). Strikingly, all the *S. islandicus* ePKs seem to be non-RD kinases due to lack of the Arg residue and may have different activation mechanisms from that for RD kinases (**Figure 1B**). It has been shown that some non-RD kinases do not autophosphorylate the activation loop and are either constitutively active or regulated by alternative mechanisms (Dardick et al., 2012). Consistently, several *in vitro* studies revealed that the phosphorylated sites of archaeal ePKs were not located within the activation loop (LaRonde-LeBlanc et al., 2005; Reimann et al., 2013; Ray et al., 2015).

The three ePKs, SiRe_0101, SiRe_2030, and SiRe_2056, are typical eukaryotic protein kinases, especially those phosphorylating eIF2α in eukaryotes. Eukaryotic eIF2α protein kinases phosphorylate eIF2α to inhibit global polypeptide synthesis in response to a variety of cellular stresses (Donnelly et al., 2013). *In vitro* kinase assay revealed that *S. solfataricus* homolog of SiRe_2056, Sso3182, was able to phosphorylate the archaeal homolog of eIF2α (aIF2α), but not on the conserved Ser51 of eIF2α (Ray et al., 2015). It was shown that the SiRe_2030 homolog in *S. acidocaldarius*, Saci_1193, may stimulate archaella expression by phosphorylating two repressors of archaella expression, ArnA and ArnB (Reimann et al., 2012). *Saci1193* was up-regulated during starvation and its deletion resulted in reduced cell motility (Hoffmann et al., 2017). In addition, *Saci_1193* was up-regulated during G1/S phase transition (Lundgren and Bernander, 2007), while the homolog in *S. solfataricus, Sso3207*, was transcriptionally repressed after UV radiation (Frols et al., 2007). In our study, SiRe_2056, but not SiRe_2030, could be overexpressed in *S. islandicus* and resulted in growth retardance. It seems that both SiRe_2030 and SiRe_2056 exhibited efficient phosphorylation ability *in vitro* and may work as master kinases. Consistently, both ePKs contain a TPR which involves in a variety of protein–protein interactions (Groves and Barford, 1999). However, they may work in different signaling pathways. We speculated that SiRe_2030 might be involved in cell cycle regulation or the responses to harsh conditions such as DNA damage for cell survival so that it could not be overexpressed, while SiRe_2056 overexpression was inducible in the presence of certain cellular stresses and a large amount of the protein with high activity would severely inhibit the cell growth. However, it should be noted that although SiRe_2056KD efficiently phosphorylated all other ePKs *in vitro*, the membrane localization would allow limited number of the substrate kinases to be accessible *in vivo*. Due to complex regulatory mechanisms under cellular conditions, the signaling pathways they transmitted may be not as same as the results based on our *in vitro* assays and need further physiological data to support. Our MS analysis of co-purified proteins of SiRe_0101KD identified many proteins that might participate in protein folding and degradation. In our previous report, only thermosomes were co-purified with a homologous recombination protein HerA using the same protocol (Huang et al., 2015). It seems that the identified proteins with high scores may be those having potential interactions with SiRe_0101 rather than unspecific binding proteins. However, the proteins with low scores in MS analysis may be contamination due to partial degraded proteins in the gel. Even though the phosphorylation activity of SiRe_0101KD on other co-purified proteins was not detectable in the autophosphorylation assay (**Figures 2B, 3A**), we could not exclude that they had already been phosphorylated after purification together with SiRe_0101KD from the host cell. It has been revealed that a *S. acidocaldarius* thermosome (SiRe_1214 homolog) and the three subunits of proteasome complex, as well as the proteasome subunits in *Haloferax volcanii*, can be phosphorylated *in vivo*, suggesting that protein folding and/or degradation were regulated by protein phosphorylation in which SiRe_0101 may be involved (Humbard et al., 2010; Reimann et al., 2013).

SiRe_1570 belongs to an ancient family of protein kinases, the piD261/Bud32 protein kinase. Studies on the homolog protein from *S. solfataricus in vitro* showed that it phosphorylated itself and some acid proteins on Ser/Thr, and its activity was stimulated by ADP-ribose (Haile and Kennelly, 2011). A protein complex KEOPS (Kinase Endopeptidase and Other Proteins of Small size), formed by Bud32, Kae1 (kinase-associated endopeptidase 1) and two small proteins, Cgi121 and Pcc1, are highly conserved throughout archaea and eukaryotes (Hecker et al., 2009). The complex is involved in the biosynthesis of the universal N^6^-threonylcarbamoyladenosine (t6A) tRNA modification (Srinivasan et al., 2011), regulation of transcription and maintenance of telomere integrity (Downey et al., 2006; Kisseleva-Romanova et al., 2006). In the *Methanocaldococcus jannaschii* genome, The Bud32 and Kae1 genes are fused into one ORF indicating they also interact with each other (Hecker et al., 2008). *M. jannaschii* and *Pyrococcus abyssi* Kae1 inhibited Bud32 autophosphorylation ability *in vitro* (Perrochia et al., 2013). The inactivation of Bud32 by Kae1 was also found in yeast (Hecker et al., 2008). The finding that overexpression of yeast Bud32 is toxic for the cell is similar to our result showing that SiRe_1570 could not be overexpressed in *S. islandicus* (**Figure 5**) (Hecker et al., 2008). Both our result of *SiRe_1570* gene deletion using CRISPR-Cas system (data not shown) and that on *S. acidocaldarius* by markerless deletion or marker insertion (Hoffmann et al., 2017) showed that a *SiRe_1570* deletion mutant could not be obtained, indicating that it might be an essential gene for cell viability. Collectively, SiRe_1570 probably has evolutionally conserved functions among archaea and eukaryotes.

SiRe_0171 (RIO1) and SiRe_1810 (RIO2) are two putative RIO protein kinases. The RIO kinase family is also ancient and found in all three domains of life. It was shown that eukaryotic RIOs participate in ribosome biogenesis, cell cycle progression, and genome integrity (Angermayr et al., 2002; Ferreira-Cerca et al., 2012; Widmann et al., 2012). *H. volcanii* RIO1 phosphorylated the α subunit of 20S proteasome on Ser and Thr sites (Humbard et al., 2010), while *S. solfataricus* RIO1 was transcriptionally induced at early stage of UV-treatment (Gotz et al., 2007). The structure of *Archaeoglobus fulgidus* RIO2 has been resolved (LaRonde-LeBlanc et al., 2005) and *P. horikoshii* RIO2 was able to phosphorylate aIF2α *in vitro* (Tahara et al., 2004). In γ–irradiation-treated *P. furiosus*, the mRNA level of RIO1 increased while that of RIO2 decreased (Williams et al., 2007). Our previous study showed that SiRe_0171 was down-regulated in *S. islandicus* treated by DNA alkylating agents methyl methanesulfonate (MMS) (Song et al., 2016). Here, we found that both RIOs displayed autophosphorylation ability and could not be overexpressed in *S. islandicus*, suggesting that they activated themselves in important cellular pathways (cell cycle regulation or DNA damage response) and their expressions were strictly regulated in the cell.

The SiRe_0181 homolog in *S. acidocaldarius* is one of the pilus components, AapE, and may participate in virus infection and the expression of *Sulfolobus* pilus (Henche et al., 2012; Deng et al., 2014). It is annotated as a secretion ATPase and its homolog in *S. solfataricus*, SsoPK2, was the only one having autophosphorylation activity among five *S. solfataricus* secretion ATPase (Lower and Kennelly, 2003; Albers and Driessen, 2005). Our results revealed that the Walker A (and/or Walker B) motif, but not the potential HRD motif identified previously, is essential for the autophosphorylation activity of SiRe_0181, indicating a different mechanism for autophosphorylation.

We showed that SiRe_1531 could not phosphorylate itself and its overexpression inhibited cell growth. Bioinformatics analysis also revealed that it is a paralog of Helicase/ATPase HerA which is involved in DNA end resection of homologous recombination (Hopkins and Paull, 2008; Huang et al., 2015). HerA overexpression led to enlarged cells with multi-chromosomes and reduced viability, probably affecting normal DNA metabolism (unpublished data). Therefore, overexpression of SiRe_1531 might have similar effect on the cell by interfering with DNA metabolism, resulting in growth retardance.

For other ePKs which did not have autophosphorylation activity, SiRe_1057, SiRe_1639, and SiRe_2600, no previous investigation was reported. In NCBI, SiRe_2600 is annotated as ABC1 kinases that might be involved in ubiquinone biosynthesis, whereas SiRe_1057 and SiRe_1639 are only annotated as Ser/Thr PKs. Our current results showed that all the ePKs were only phosphorylated by heterologous ePKs *in vitro* and each can be overexpressed in *S. islandicus*, indicating that they might locate at the bottom of the hierarchy network which needs to be activated by upstream ePKs.

**FIGURE 6:**
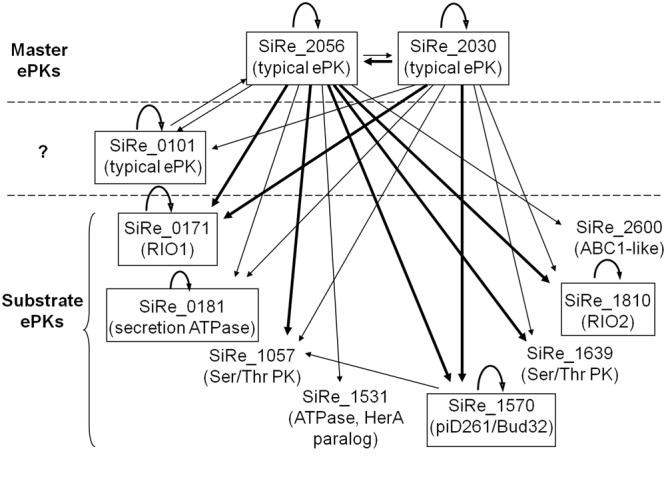
A proposed regulatory network of *S. islandicus* ePKs according to auto- and cross-phosphorylation results. The two putative master regulator kinases are SiRe_2030 and SiRe_2056 which exhibit autophosphorylation activities and efficiently phosphorylate on most of the other ePKs. In addition, SiRe_0101 contains TM domain and phosphorylates SiRe_2056KD *in vitro*, suggesting that it may work as an accessory kinase for the master kinase that responds to an extracellular signal and spread the signal through SiRe_2056. On the other hand, the substrate kinases are those that do not transfer phosphates to the other ePKs. The ePKs having autophosphorylation activities are indicated in boxes. The arrow indicates the direction of phosphorylation, in which the thick lines represent high activities. The names of the most closely related protein kinase subfamily are shown in the parentheses (see the text for details).

Based on the phosphorylation of the ePKs on themselves and the other ePKs, we propose a putative regulatory network of *S. islandicus* ePKs (**Figure 6**). It is composed of two distinct functional classes: master regulator kinases and substrate kinases. SiRe_2030 and SiRe_2056 exhibited higher autophosphorylation activities and phosphorylation on other ePKs and therefore can be defined as the master regulator kinases. Since SiRe_0101 also contains a TM domain and phosphorylates SiRe_2056KD, it may serve as an accessory kinase for the master kinase sensing the extracellular signals. The base of the regulatory network is built up by the substrate kinases that were phosphorylated by master kinases and do not have phosphorylation activity on other ePKs. The fact in the network that one master ePK is able to phosphorylate a number of substrate ePKs while a substrate ePK can also be phosphorylated by several master ePKs (mainly by SiRe_2030 and SiRe_2056) is similar to those in eukaryotes and bacteria. However, only two regulatory layers exist in *S. islandicus* ePK network, suggesting that it is simpler than those in the other two domains, which have multiple-layered regulatory network. In addition, it seems that the typical ePKs, which emerged later than the ancient RIOs and Bud32 kinases, become the master kinases. We speculate that the RIOs and Bud32 are ancient and evolutionarily conserved kinases which should have conserved functions *in vivo*. While during a long time of evolution, species need new strategies for responding to new environments and survival. That could be the reason why the ePKs like SiRe_0101, SiRe_2056 and SiRe_2030 emerged, which contain a TM domain for membrane localization and sensing the extra-cellular signals and/or a TPR that mediate protein–protein interactions. Consistently, both master kinases in *M. tuberculosis*, PknB and PknH, contain TM and folded extracellular sensor domains (Baer et al., 2014). In addition, the low conservation of these ePKs in archaea (SiRe_0101 and SiRe_2056 even do not exist in several *Sulfolobus* species) is in agreement with our speculation that they emerged later to sense various signals in different environments. The autophosphorylation ability of the RIOs and Bud32 kinases also indicates that these kinases may not completely depend on the master kinases, but activate themselves in some essential conserved pathways.

## Author Contributions

QH designed the project, conducted most of the experiments, analyzed the data, and wrote draft of the paper. QZ and JM performed part of the experiments in plasmid construction and protein purification. JM and JN helped revised the manuscript. YS conceived the idea for the project and helped write the paper. All authors approved the version to be published and agreed to be accountable for all aspects of the work in ensuring that questions related to the accuracy or integrity of any part of the work are appropriately investigated and resolved.

## Conflict of Interest Statement

The authors declare that the research was conducted in the absence of any commercial or financial relationships that could be construed as a potential conflict of interest.

## Abbreviations

ABC1: activator of bc1 complex
ePK: eukaryotic-like protein kinase
eSTK: eukaryotic-like Ser/Thr kinase
HRD: His-Arg-Asp
KD: protein kinase catalytic domain
PTP: protein tyrosine phosphatase
RIO: right open reading frame
TM: transmembrane
TPR: tetratricopeptide repeat
wHTH: winged helix-turn-helix
