# Supplementary material for: Autophosphorylation and Cross-Phosphorylation of Protein Kinases from the Crenarchaeon Sulfolobus islandicus
Source: Front Microbiol. 2017 Nov 7;8:2173. doi: 10.3389/fmicb.2017.02173 (PMC5682000; doi:10.3389/fmicb.2017.02173)
Supplement: Supplementary file 1 [file Table_1.docx]

**Table S1. Oligonucleotides used in this study**

| **Primer** | **Sequence ^a^ (5’-3’)** |
| --- | --- |
| 0101KD-NdeI-F | CGAGGAC**CATATG**AAGATAAATGAATACGAAG |
| 0101-SalI-R | GCGG**GTCGAC**TTAACGTAAATTATATTCTAT |
| 0101-Nostop-SalI-R | GCGC**GTCGAC**ACGTAAATTATATTCTATAG |
| 0101-D393N-F | CTGGTTATGTTCATTTAAATGTAAAACCACAAAAT |
| 0101-D393N-R | ATTTTGTGGTTTTACATTTAAATGAACATAACCAG |
| 0171-NdeI-F | CAACGCC**CATATG**ACGGAATTACCAAAG |
| 0171-SalI-R | AATT**GTCGAC**TCAATCCCCGGAAACTC |
| 0171-Nostop-SalI-R | CATA**GTCGAC**ATCCCCGGAAACTCCGAA |
| 0171-D188N-F | GCTAAATTGGTTCACGGAAACTTAAGTGAGTAT |
| 0171-D188N-R | ATACTCACTTAAGTTTCCGTGAACCAATTTAGC |
| 0181-NdeI-F | CCGCCGC**CATATG**AGTAAAATTTTTAAATCTC |
| 0181-SalI-R | CGCC**GTCGAC**TTATTCTTTTAATTCTATAGCT |
| 0181-Nostop-SalI-R | GGCC**GTCGAC**TTCTTTTAATTCTATAGCTTTT |
| 0181-D490N-F | GTTGGGATCCGAAAAGTAACACATTTACAATC |
| 0181-D490N-R | GATTGTAAATGTGTTACTTTTCGGATCCCAAC |
| 0181-NdeI-M-F | TTACGCTAGACCAGCTTATGCAGGAGTA |
| 0181-NdeI-M-R | TACTCCTGCATAAGCTGGTCTAGCGTAA |
| 1057-NdeI-F | CGGGCTG**CATATG**GTTAAAATAAGCTGTT |
| 1057-SalI-R | CCAT**GTCGAC**TTAGAACAACTGAGATAAG |
| 1057-Nostop-SalI-R | CATA**GTCGAC**GAACAAC TGAGATAAGAGC |
| 1057-E135Q-F | CTCATAGAGCATAAACAATTGAGTAGACC |
| 1057-E135Q-R | GGTCTACTCAATTGTTTATGCTCTATGAG |
| 1531-NdeI-F | CGCCCCG**CATATG**ATGGAAAGTATTTTTG |
| 1531-SalI-R | GCCC**GTCGAC**TCATAGCTTATTTATTCTATC |
| 1531-Nostop-SalI-R | CGCC**GTCGAC**TAGCTTATTTATTCTATCCT |
| 1531-D349N-F | CAGTGGATGATATCATAAACGACTCTATTAATAATT |
| 1531-D349N-R | AATTATTAATAGAGTCGTTTATGATATCATCCACTG |
| 1570-NdeI-F | GGATGCT**CATATG**GAGAGTAGACGAGGTAG |
| 1570-SalI-R | CCAT**GTCGAC**TTACTCGTTACCACTCCTAT |
| 1570-Nostop-SalI-R | GACT**GTCGAC**CTCGTTACCACTCCTATTC |
| 1570-D134N-F | GGGATAGCTCACGGTAATCTAACAACTAACAA |
| 1570-D134N-R | TTGTTAGTTGTTAGATTACCGTGAGCTATCCC |
| 1639-NdeI-F | GGCGTTA**CATATG**ATGTTTAAAAATTCATACAG |
| 1639-BamHI-F | CGCC**GGATCC**CATGTTTAAAAATTCATACA |
| 1639-SalI-R | GGCC**GTCGAC**TTAATAAAAATTACTCATTA |
| 1639-Nostop-SalI-R | GGCG**GTCGAC**ATAAAAATTACTCATTAAGT |
| 1639-D117A-F | ATATGTGCTAGGTGCCACTAAAATAAGTAA |
| 1639-D117A-R | TTACTTATTTTAGTGGCACCTAGCACATAT |
| 1639-NdeI-M-F | TTTGGAATTATCCGTATGTTGCTGATCC |
| 1639-NdeI-M-R | GGATCAGCAACATACGGATAATTCCAAA |
| 1810-NdeI-F | TTTACGA**CATATG**AGACTTTCACTGGCGG |
| 1810-SalI-R | GATC**GTCGAC**TCATGATACCCCTCTTACA |
| 1810-Nostop-SalI-R | GAAG**GTCGAC**TGATA CCCCTC TTACATAA |
| 1810-D227N-F | CATAACACATGGAAATCTAAGCCCGTATAATG |
| 1810-D227N-R | CATTATACGGGCTTAGATTTCCATGTGTTATG |
| 2030-NdeI-F | CGGG**CATATG**GTGCAGTTAGTATTACAGTT |
| 2030-SalI-R | GTAC**GTCGAC**TCAATAGCTTATAAGCTTTTCTGC |
| 2030-Nostop-SalI-R | GTGGCTG**GTCGAC**ATAGCTTATAAGCTT |
| 2030-D498A-F | GGGTATACGCATTGTGCCATAAAACCATCT |
| 2030-D498A-R | AGATGGTTTTATGGCACAATGCGTATACCC |
| 2056KD-NdeI-F | GCCTCG**CATATG**TCACCAGAGATGATATATT |
| 2056-SalI-R | GGCG**GTCGAC**TTATATCTTGGAATAGAAAAAC |
| 2056-Nostop-SalI-R | GGCG**GTCGAC**TATCTTGGAATAGAAAAAC |
| 2056-D476A-F | GGCTACGTTCACTGTGCTATTAAACCTCAA |
| 2056-D476A-R | TTGAGGTTTAATAGCACAGTGAACGTAGCC |
| 2056-NdeI-M-F | GTTATACTCCAGCCTATGTACCATTTGAC |
| 2056-NdeI-M-R | GTCAAATGGTACATAGGCTGGAGTATAAC |
| 2600-NdeI-F | CGGCGTC**CATATG**ATTAAAAGATTACTGAAAG |
| 2600-SalI-R | TCGT**GTCGAC**TTATGTATTTTTTCTTCCGC |
| 2600-Nostop-SalI-R | TCGT**GTCGAC**TGTATTTTTTCTTCCGCTAAG |
| 2600-D262N-F | GACTATTTTCATGCAAATCCTCACCCTGG |
| 2600-D262N-R | CCAGGGTGAGGATTTGCATGAAAATAGTC |
| 2600-NdeI-M-F | AACTTATTGATTAGGGCCTATGTTGCAATGA |
| 2600-NdeI-M-R | TCATTGCAACATAGGCCCTAATCAATAAGTT |
| 0241-NdeI-F | GATCGAG**CATATG**TATTGGGTTAGAAGGAA |
| 0241-SalI-R | ACGT**GTCGAC**CTAAGAATTCGAGTAGATG |
| 0241-NdeI-M-F | GCAGTACAAACATACGAGCAAGAGATGTTC |
| 0241-NdeI-M-R | GAACATCTCTTGCTCGTATGTTTGTACTGC |
| 1009-NdeI-F | GCTGCCG**CATATG**ATGAATTTAGATGAAGTTA |
| 1009-SalI-R | AGTC**GTCGAC**CTAAACATACGCGTAATCAAA |

^a^ Restriction sites are indicated in boldface, mutated codons for construction of site-directed mutants are underlined and mutated codons in the NdeI restriction site of kinase genes are indicated in box.
