# Supplementary material for: Autophosphorylation and Cross-Phosphorylation of Protein Kinases from the Crenarchaeon Sulfolobus islandicus
Source: Front Microbiol. 2017 Nov 7;8:2173. doi: 10.3389/fmicb.2017.02173 (PMC5682000; doi:10.3389/fmicb.2017.02173)
Supplement: Supplementary file 2 [file Table_2.docx]

**Table S2. Plasmids used in this study**

| **Plasmids** | **Features** | **Source or reference** |
| --- | --- | --- |
| pSeSD | *E. coli/Sulfolobus* shuttle vector harboring the marker *pyrEF* | Peng, *et al*., 2012. |
| pET15b-N-His-0171,  pET15b-N-His-0181,  pET15b-N-His-1057,  pET15b-N-His-1531,  pET15b-N-His-1570,  pET15b-N-His-1810,  pET15b-N-His-2030,  pET15b-N-His-2056KD,  pET15b-N-His-2600 | pET15b carrying SiRe_0171, 0181, 1057, 1531, 1570, 1810, 2030, 2056KD or 2600 gene | This work |
| pRSFDuet-1-N-His-1639,  pRSFDuet-1-N-His-1639D117A | pRSFDuet-1 carrying SiRe_1639 or 1639D117A gene | This work |
| pET15b-N-His-0171D188N,  pET15b-N-His-0181D490N,  pET15b-N-His-1057E135Q,  pET15b-N-His-1531D349N,  pET15b-N-His-1570D134N,  pET15b-N-His-1810D227N,  pET15b-N-His-2030D498A,  pET15b-N-His-2056KD-D476A,  pET15b-N-His-2600D262N | pET15b carrying SiRe_0171 D188N, 0181 D490N, 1057 E135Q, 1531 D349N, 1570 D134N, 1810 D227N, 2030 D498A, 2056KD-D476A or 2600 D262N gene | This work |
| pET15b-N-His-0241,  pET15b-N-His-1009 | pET15b carrying SiRe_0241 and 1009 gene | This work |
| pSeSD-0101KD-C-His,  pSeSD-0171-C-His,  pSeSD-0181-C-His,  pSeSD-1057-C-His,  pSeSD-1531-C-His,  pSeSD-1570-C-His, pSeSD-1639-C-His,  pSeSD-1810-C-His,  pSeSD-2030-C-His, pSeSD-2056KD-C-His,  pSeSD-2600-C-His | pSeSD carrying SiRe_0101KD, 0171, 0181, 1057, 1531, 1570, 1639, 1810, 2030, 2056KD or 2600 gene | This work |
