# Supplementary material for: Autophosphorylation and Cross-Phosphorylation of Protein Kinases from the Crenarchaeon Sulfolobus islandicus
Source: Front Microbiol. 2017 Nov 7;8:2173. doi: 10.3389/fmicb.2017.02173 (PMC5682000; doi:10.3389/fmicb.2017.02173)
Supplement: Supplementary file 4 [file Data_Sheet_1.docx]

Supplementary Material

**Autophosphorylation and Cross-phosphorylation of Protein Kinases from the Crenarchaeon *Sulfolobus islandicus***

**Qihong Huang, Qing Zhong, Joseph Badys Amozay Mayaka, Jinfeng Ni, Yulong Shen***

***Correspondence**: Yulong Shen: [yulgshen@sdu.edu.cn](mailto:yulgshen@sdu.edu.cn)

# Supplementary Figures and Tables

## Supplementary Figures


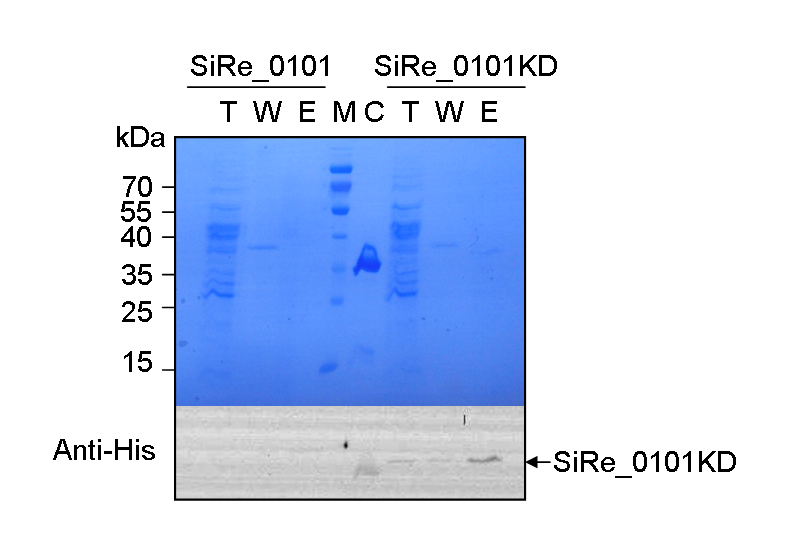


**Supplementary Figure 1.** Western blot analysis of SiRe_0101-C-His and SiRe_0101KD-C-His purified from *S. islandicus.* SiRe_0101-C-His (63.1 kDa) and SiRe_0101KD-C-His (35.8 kDa) were induced in their corresponding overexpression strains with arabinose. Cells were disrupted and the soluble proteins were applied to a Ni-NTA column for purification. The fractions of total proteins (T), washed (W), and eluted samples (E) were analyzed by Western blot using anti-His-tag antibody. M, protein marker. C, control (purified N-His-SiRe_1810 (33.0 kDa) from *E. coli*).


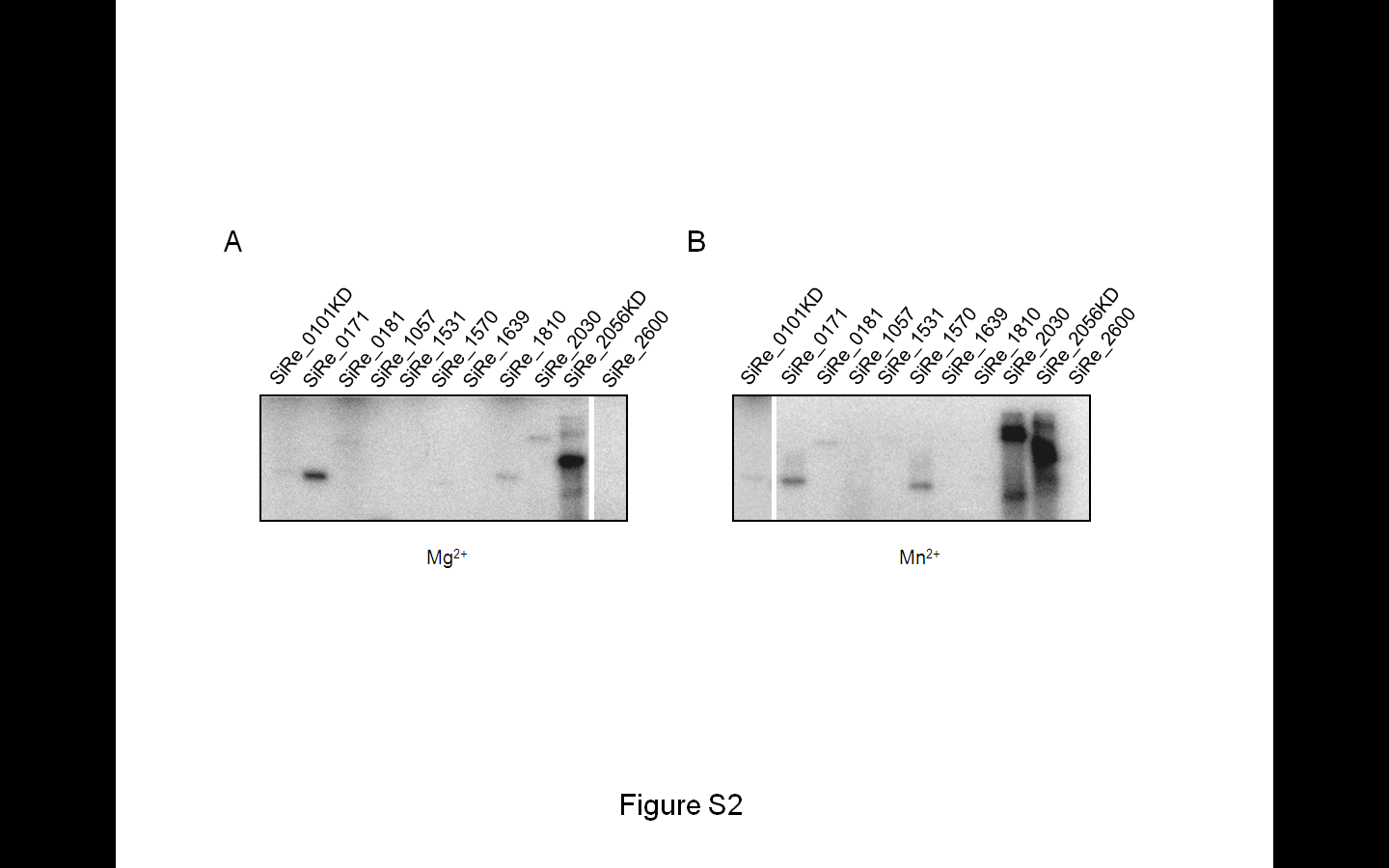


**Supplementary Figure 2.** Autophosphorylation activities of *S. islandicus* ePKs in the presence of Mg^2+^ or Mn^2+^. Each ePK was incubated with 4.2 nM [γ-^32^P]ATP and 50 μM cold carrier ATP in a reaction containing Mg^2+^ (A) or Mn^2+^ (B) at 65^o^C (see the text). The samples were analyzed by 15% SDS-PAGE. The experiment was performed at least for three times. Representative images of ePKs autophosphorylation activities are shown.


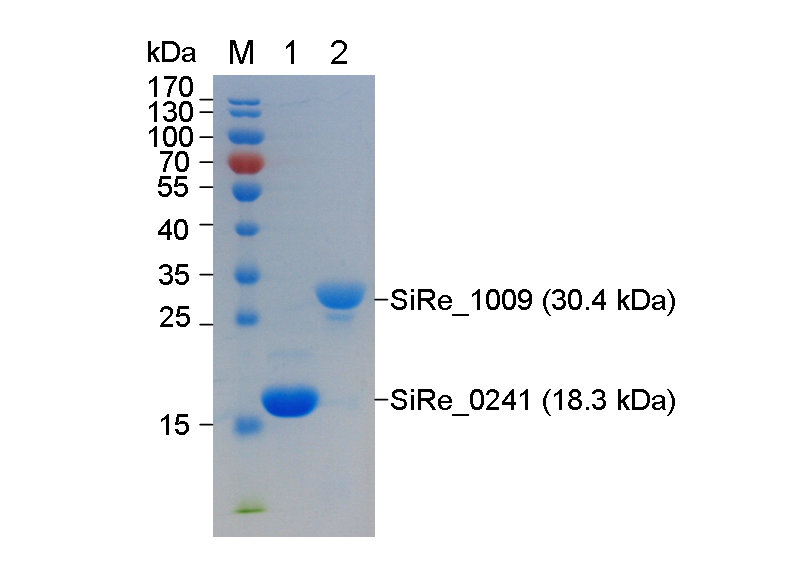


**Supplementary Figure 3.** Purification of two protein phosphatase, SiRe_0241 and SiRe_1009. The two proteins purified with a Ni-NTA column and subsequent gel filtration were analyzed by SDS-PAGE. For more detail, please see the Materials and Methods in the text. M, protein size marker; 1, SiRe_0241 (18.3 kDa); 2, SiRe_1009 (30.4 kDa).


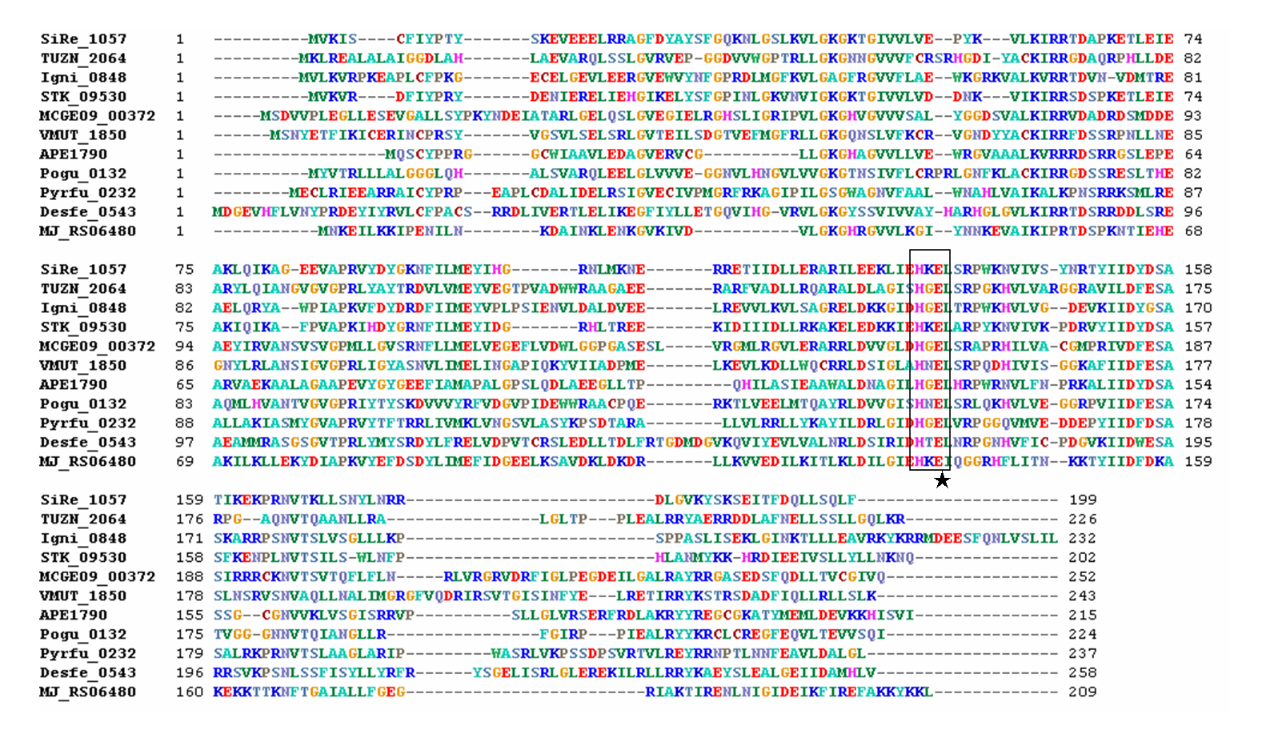


**Supplementary Figure 4.** Alignment of archaeal SiRe_1057 homologs. The alignment is performed by Bioedit. The highly conserved Glu (E) instead of Asp (D) within the HRD motif of subdomain VIb (the dashed box) is indicated with the star. TUZN, *Thermoproteus uzoniensis*; Igni, *Ignicoccus hospitalis*; STK, *Sulfolobus tokodaii*; MCGE09, *Thaumarchaeota archaeon*; VMUT, *Vulcanisaeta moutnovskia*; APE, *Aeropyrum pernix*; Pogu, *Pyrobaculum oguniense*; Pyrfu, *Pyrolobus fumarii*; Desfe, *Desulfurococcus fermentans*; MJ, *Methanocaldococcus jannaschii*.


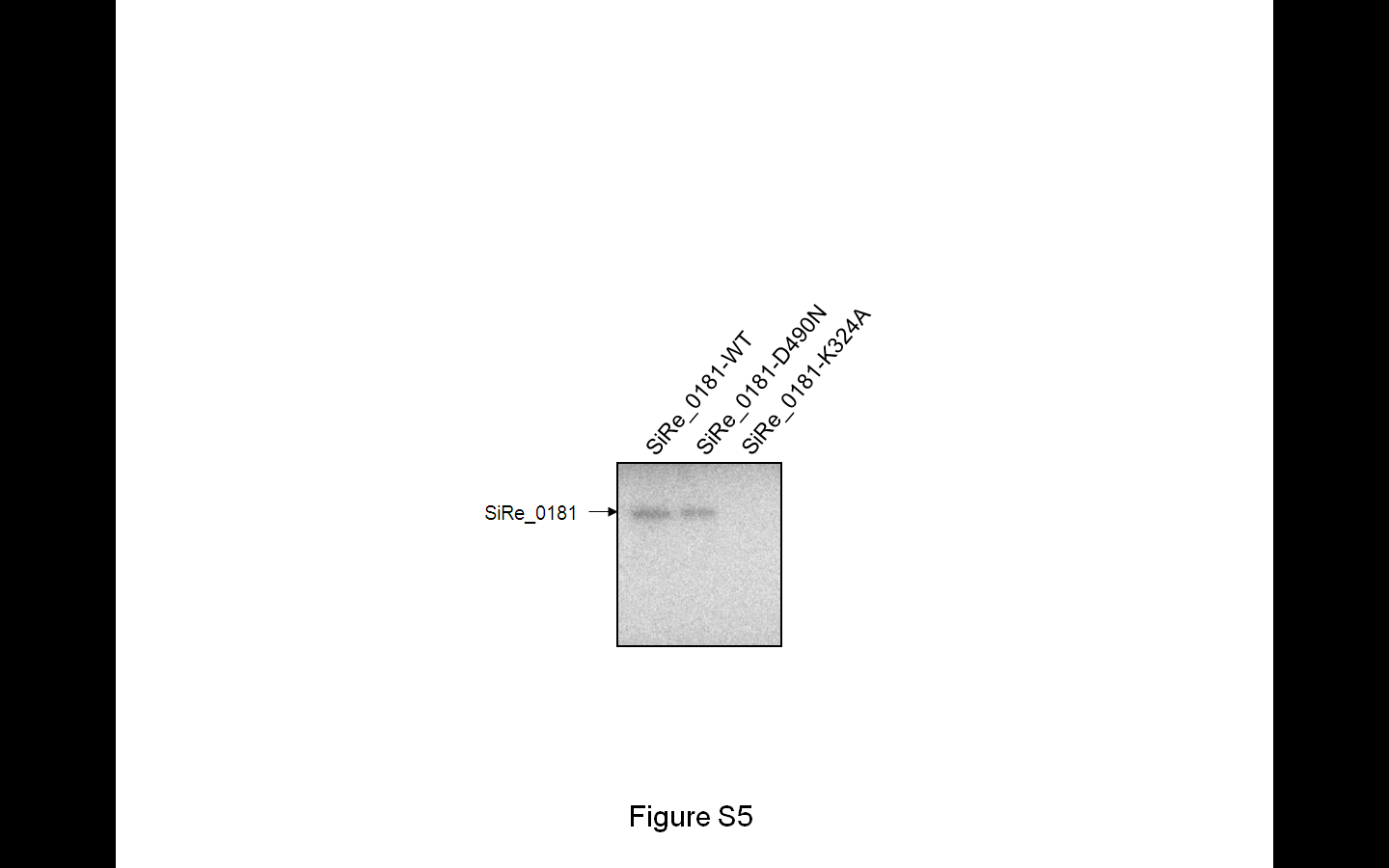


**Supplementary Figure 5.** SiRe_0181K324A (a mutation in conserved Walker A motif) but not SiRe_0181D490N (a mutation in the potential HRD motif) loses its autophosphorylation activity. An amount of 1 μM wild type (WT) SiRe_0181, SiRe_0181-D490N, or SiRe_0181-K324A was used in the autophosphorylation assay.
